# Complete mitochondrial genome and the phylogenetic position of the graceful catshark *Proscyllium habereri* (Carcharhiniformes: Proscylliidae)

**DOI:** 10.1080/23802359.2016.1159934

**Published:** 2016-03-29

**Authors:** Hao Chen, Junqi Yu, Ranran Si, Xiao Chen, Weiming Ai

**Affiliations:** a Department of Marine Biotechnology, School of Life Science, Wenzhou Medical University, Wenzhou, Zhejiang, PR China;; b Zhejiang Mariculture Research Institute, Wenzhou, Zhejiang, PR China

**Keywords:** Mitochondrial genome, Proscylliidae, *Proscyllium habereri*

## Abstract

In this study, the complete mitogenome of *Proscyllium habereri* (Carcharhiniformes: Proscylliidae) is first determined. It is 16,708 bp in length, containing 37 genes with typical order to that of most other vertebrates. Its overall base composition of the H-strand is A 30.9%; C 23.7%; G 14.2%; T 31.2%. Two start codons (ATG and GTG) and two stop codons (TAG and TAA/T) are found in the protein-coding genes. The 22 tRNA genes range from 67 bp (tRNA-*Cys*, tRNA-*Ser*2) to 75 bp (tRNA-*Leu*1). The phylogenetic result showed that *P. habereri* was clustered to *Pseudotriakis microdon*.

The graceful catshark *Proscyllium habereri* (Carcharhiniformes: Proscylliidae) is a little-known, uncommon bottom-dwelling shark found on the shelves of continental and insular waters of Western Pacific, and it’s also an oviparous fish, with a single egg deposited per uterus (Compagno 1984; Compagno & Niem 1998). In this study, we first determine the complete mitogenome of *P. habereri*, the first species in family Proscylliidae uploaded to genebank, and analyzed the phylogenetic relationship of the sharks in Carcharhiniformes.

One specimen of *P. habereri* (Museum of marine biology of Wenzhou Medical University, voucher DS2011061714) was captured in Dongshan Bay, Fujian province, China. The experimental protocol and data analysis methods followed Chen et al. (2014). Including *P. habereri*, 30 species of Carcharhiniformes with complete mitogenomes available in the GenBank were selected to construct the phylogenetic tree. The Bayesian method was fulfilled with the GTR + I + G model by four partitions of the mitogenomic data: 12S and 16S rRNA genes, the first and second codons of the 12 protein-coding genes (except *ND6* gene), and 14 tRNA genes located in heavy chain of mitochondrial DNA.

The total length of the complete mitochondrial genomes of *P. habereri* is 16,708 bp (Genbank accession no. KU721838). It contains 13 protein-coding genes, 22 tRNA genes, two rRNA genes and one non-coding control region, with the typical gene composition, arrangement and transcriptional orientation in most mitogenomes of vertebrates. The overall base composition of the H-strand is A 30.9%; C 23.7%; G 14.2%; T 31.2%. Except for *COI* gene, which starts with the GTG codon as common in vertebrates (Slack et al. 2003), all protein-coding genes use the standard ATG codon as initiation codon and the typical TAG and TAA/T codon as terminal codon. Both 12S rRNA (951 bp) and 16S rRNA (1668 bp) genes are between tRNA-*Phe* and tRNA-*Leu*1 genes, separated by tRNA-*Val* gene. Twenty-two tRNA genes intersperses between the rRNAs and protein-coding genes, ranging from 67 bp (tRNA-*Cys*, tRNA-*Ser*2) to 75 bp (tRNA-*Leu*1). All tRNAs can fold into a typical clover-leaf secondary structure, except the tRNA-*Ser*2 replacing the dihydrouridine arm by a simple loop. The origin of L-strand replication (38 bp) is identified between tRNA-*Asn* and tRNA-*Cys* genes, which can fold a hairpin structure (12-bp stem and 14-bp loop) as a signal to initiate the replication of L-strand. The control region (1067 bp) is located between the tRNA-*Pro* and tRNA-*Phe* genes.

Seven families of Carcharhiniformes are included in the phylogenetic tree. Most nodes of the Bayesian tree are well supported (Figure 1). The relationship of four basal families (Scyliorhinidae, Pseudotriakidae, Proscylliidae and Triakidae) is clear. *Proscyllium habereri* is clustered to *Pseudotriakis microdon* with high support value (100%). In addition, two monophyletic families Sphyrnidae and Hemigaleidae are embedded between *Galeocerdo cuvier* (Carcharhinidae) and the remaining Carcharhinidae species, which is consistent with the prior molecular phylogenetic result that constructed by one nuclear (*Rag1*) and four mitochondrial (*COI*, *ND2*, *Cytb* and *16S*) genes (Vélez-Zuazo et al. 2011). It suggests that the current family Carcharhinidae is paraphyletic, and the taxonomy of *G. Cuvier* need more study.

**Figure 1.**
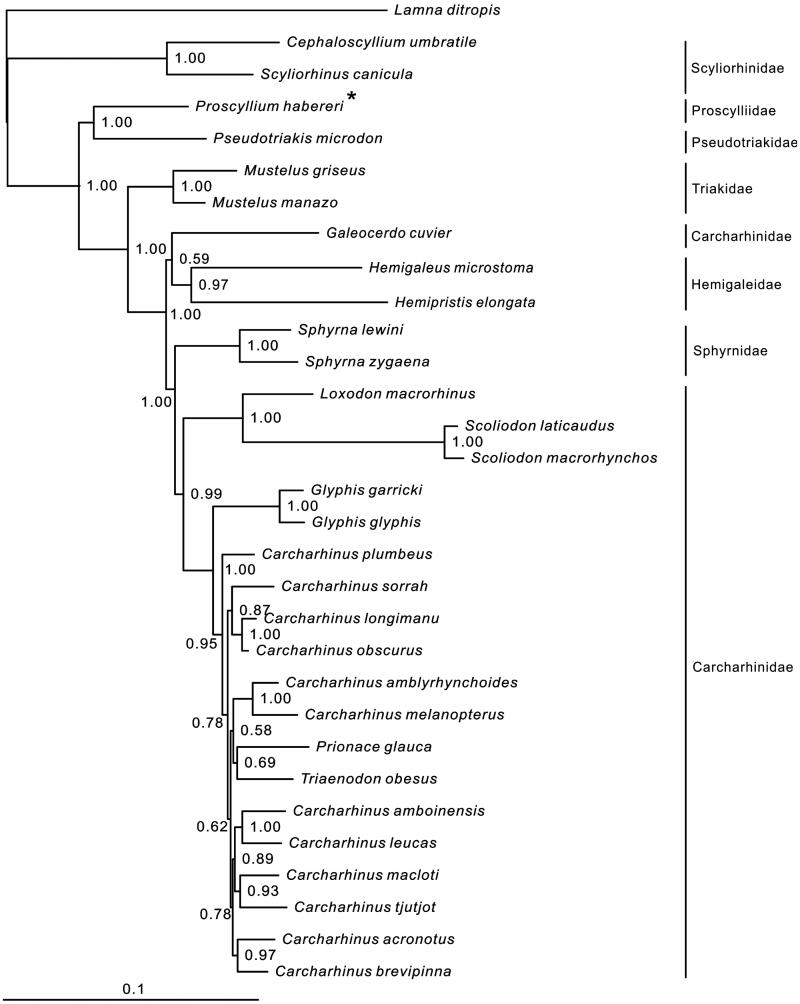
Phylogenetic position of *Proscyllium habereri Lamna ditropis* (KF962053.1) was selected as the out group. The 29 species from the order Carcharhiniformes were: *Carcharhinus acronotus* (NC_024055.1), *C. amblyrhynchoides* (NC_023948.1), *C. amboinensis* (NC_026696.1), *C. brevipinna* (KM244770.1), *C. leucas* (KF646785.1), *C. longimanu* (NC_025520.1), *C. macloti* (NC_024862.1), *C. melanopterus* (NC_024284.1), *C. obscurus* (NC_020611.1), *C. plumbeus* (NC_024596.1), *C. sorrah* (NC_023521.1), *C. tjutjot* (KP091436.1) *Galeocerdo cuvier* (NC_022193.1), *Loxodon macrorhinus* (KT347599), *Prionace glauca* (NC_022819.1), *Scoliodon laticaudus* (KP336547.1)*, S. macrorhynchos* (NC_018052.1), *Triaenodon obesus* (KJ748376.1), *Glyphis glyphis* (NC_021768.2), *G. garricki* (KF646786.1), *Mustelus griseus* (NC_023527.1), *M. manazo* (NC_000890.1), *Cephaloscyllium umbratile* (KT003686), *Hemigaleus microstoma* (KT003687), *Hemipristis elongata* (KU508621), *Scyliorhinus canicula* (NC_001950.1), *Pseudotriakis microdon* (NC_022735.1), *Proscyllium habereri* (KU721838*), Sphyrna lewini* (NC_022679.1), *Sphyrna zygaena* (NC_025778.1).
